# Clinical outcomes in estrogen receptor-positive early-stage breast cancer patients with Recurrence Score 26-30: observational real-world cohort study

**DOI:** 10.1038/s41523-023-00549-8

**Published:** 2023-06-02

**Authors:** Ofer Rotem, Idit Peretz, Michelle Leviov, Iryna Kuchuk, Amit Itay, Margarita Tokar, Shani Paluch-Shimon, Ofra Maimon, Rinat Yerushalmi, Karen Drumea, Ella Evron, Amir Sonnenblick, Einav Gal-Yam, Hadar Goldvaser, Yosef Samih, Rotem Merose, Avital Bareket-Samish, Lior Soussan-Gutman, Salomon M. Stemmer

**Affiliations:** 1 Davidoff Center, Rabin Medical Center, Petah Tikva, Israel; 2 Lin Medical Center, Haifa, Israel; 3 Oncology Dept., Meir Medical Center, Kfar Saba, Israel; 4 Oncology Dept., Sheba Medical Center, Ramat Gan, Israel; 5 Department of Oncology, Soroka University Medical Center and the Faculty of Health Sciences, Ben-Gurion University of the Negev, Beer Sheva, Israel; 6 Sharett Institute of Oncology, Hadassah-Hebrew University Medical Center, Jerusalem, Israel; 7 Faculty of Medicine, Hebrew University, Jerusalem, Israel; 8 Sackler Faculty of Medicine, Tel Aviv University, Tel Aviv, Israel; 9 Oncology Dept., Kaplan Medical Center, Rehovot, Israel; 10 Oncology Dept., Tel-Aviv Sourasky Medical center, Tel Aviv, Israel; 11 Oncology Institute, Shaare Zedek Medical Center, Jerusalem, Israel; 12 Oncology Dept., Ha’emek Medical Center, Afula, Israel; 13 Oncology Dept., Shamir (Assaf Harofeh) Medical Center, Zerifin, Israel; 14 BioInsight Ltd, Binyamina, Israel; 15 Oncotest-Rhenium, Modi’in, Israel

**Keywords:** Breast cancer, Chemotherapy

## Abstract

Data on adjuvant chemotherapy (CT) benefit in ER + HER2‒ early-stage breast cancer (EBC) patients with Recurrence Score (RS) 26-30 are limited. This real-world study evaluated the relationships between the RS, adjuvant treatments, and outcomes in 534 RS 26-30 patients tested through Clalit Health Services (N0: *n* = 394, 49% CT-treated; N1mi/N1: *n* = 140, 62% CT-treated). The CT-treated and untreated groups were imbalanced (more high-risk clinicopathologic characteristics in CT-treated patients). With median follow-up of 8 years, Kaplan–Meier estimates for overall survival (OS), distant recurrence-free survival (DRFS), and BC-specific mortality (BCSM) were not significantly different between CT-treated and untreated N0 patients. Seven-year rates (95% CI) in CT-treated vs untreated: OS, 97.9% (94.4–99.2%) vs 97.9% (94.6–99.2%); DRFS, 91.5% (86.6–94.7%) vs 91.2% (86.0–94.6%); BCSM, 0.5% (0.1–3.7%) vs 1.6% (0.5–4.7%). For N1mi/N1 patients, OS/DRFS did not differ significantly between treatment groups; whereas BCSM did (1.3% [0.2–8.6%] vs 6.2% [2.0–17.7%] for CT-treated and untreated patients, respectively, *p* = 0.024).

## Introduction

The 21-gene Oncotype DX Breast Recurrence Score^®^ assay is a prospectively validated prognosticator and predictor of chemotherapy (CT) benefit, and is used to guide adjuvant treatment decisions in patients with early stage hormone receptor-positive human epidermal growth factor receptor 2 negative (HER2‒) breast cancer (BC)^1–7^.

The initial validation of the 21-gene assay in N0 patients, which used a prospective-retrospective study design, demonstrated that in N0 patients with Recurrence Score^®^ (RS) ≤ 30, adjuvant chemoendocrine therapy (CET) was not superior to endocrine therapy (ET) alone^8^. The prospective phase 3 TAILORx trial confirmed non-inferiority of ET vs CET in N0 patients with RS 11–25, although the study suggested that adjuvant CT may confer some benefit for some patients ≤50 years^1,2^. Similarly, the initial validation in node-positive patients using the prospective-retrospective design showed no apparent benefit for CET over ET alone in patients with RS ≤ 30^9^. The recent prospective phase 3 RxPONDER trial confirmed that for postmenopausal BC patients with 1–3 positive nodes (N1) and RS 0-25, CET was not superior to ET alone, whereas for premenopausal patients with N1 disease and RS 0-25, adding CT resulted in improved invasive disease-free survival (IDFS) and distant recurrence-free survival (DRFS)^3^. Thus, data regarding the potential benefit of CT in N0 and N1 patients with RS 26-30 are limited, as are data on patients with micrometastases (N1mi), since the TAILORx trial excluded N1mi patients, whereas data on such patients in the RxPONDER trial have not been published^1–3^.

In Israel, the 21-gene assay has been available since 2004. Since the start of the reimbursement of the assay by Clalit Health Services (CHS; the largest health maintenance organization in Israel with approximately 4 million members) in 2006, data on assays performed through CHS were collected in a registry. Various analyses of the registry data have been published thus far, including 5- and 10-year clinical outcome data^10–13^.

The current exploratory analysis of the mature CHS registry evaluated a cohort of patients with N0/N1mi/N1 BC and RS 26–30 whose treatment decisions in real-world clinical practice incorporated the RS results, and thus, it is a focused extension of prior published CHS registry analyses that examined the entire RS range^11–13^. The objectives of the current analyses were to evaluate the relationships between the RS results (within the 26–30 range), adjuvant treatments received, and clinical outcomes.

## Results

### Patient characteristics, RS, and treatments received

**Fig. 1:**
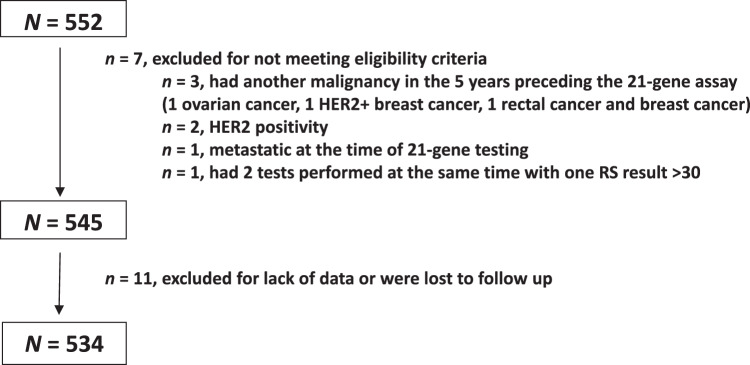
Patient disposition. HER2 human epidermal growth factor receptor 2, RS Recurrence Score.

Overall, 552 patients were tested and had RS 26–30 in the defined timeframe, of whom 18 were excluded from the current analysis. Eleven patients had no treatment or follow-up data, 3 had another malignancy in the 5 years preceding the 21-gene assay (1 ovarian cancer, 1 HER2 + BC, and 1 rectal cancer and BC), 2 were HER2 + , 1 was metastatic at the time of testing, and 1 had 2 tests performed at the same time of which one yielded RS result >30 (Fig. 1). Thus, the final cohort included 534 patients of whom 394 (73.8%) had N0 disease and 140 (26.2%) had N1mi/N1 disease.

Patient and tumor characteristics of the 394 N0 patients and 140 N1mi/N1 patients overall and by treatment received (CT vs no CT) are detailed in Table 1. Most patients received ET (498 patients, 93.3%). Four patients (0.7%) did not receive ET, and ET information was unavailable for 32 patients (6.0%). Of the N0 patients, 194 (49.2%) received CT and of the N1mi/N1 patients, 87 (62.1%) received CT. In the N0 patients, the median (range) age for all patients was 61 (28–84) years, the majority of patients (84.8%) had invasive ductal carcinoma (IDC), approximately half (50.3%) had grade 2 tumors, and the tumor size in the majority of patients (53.3%) was >1-2 cm. The N0 CT-treated and untreated patients differed significantly with respect to age at diagnosis (younger patients in the CT-treated group), and tumor size (larger tumors in the CT-treated group). Notably, of the 200 patients not treated with CT, 2 (1.0%) did not receive ET, and for 19 (9.5%) ET information was not available; of the 194 CT-treated patients, 1 (0.5%) did not receive ET and ET information was not available for 13 (6.7%) patients. This difference in ET treatment was not statistically significant (*p* = 0.504). In the N1mi/N1 patients, the median (range) age for all patients was 62 (36–85) years, the majority of patients (87.9%) had IDC, grade 2 was reported for less than half of the patients (45.0%), and the tumor size in approximately half of the patients (48.6%) was >1–2 cm. Among the N1mi/N1 population, the only statistically significant difference between CT-treated and untreated patients was age (median age and age distribution), with younger patients in the CT-treated group. Notably, all the 53 patients not treated with CT received ET; of the 87 CT-treated patients, 1 (1.1%) did not receive ET. This difference in ET treatment was not statistically significant (*p* = 0.435).

**Table 1:** Baseline patient and tumor characteristics.

	N0 Patients (*n* = 394)				N1mi/N1 Patients (*n* = 140)			
	No CT *n* = 200	CT *n* = 194	All *n* = 394	*p*-value^1^	No CT *n* = 53	CT *n* = 87	All *n* = 140	*p*-value^1^
Sex, *n* (%)								
Female	197 (98.5%)	193 (99.5%)	390 (99.0%)	0.318	50 (94.3%)	84 (96.6%)	134 (95.7%)	0.537
Median (range) age, years	64 (37–84)	58 (28–78)	61 (28–84)	<0.001	66.5 (36–85)	58 (38–77)	62 (36–85)	<0.001
Age category, *n* (%)								
<40 years	3 (1.5%)	8 (4.1%)	11 (2.8%)	<0.001	1 (1.9%)	2 (2.3%)	3 (2.1%)	<0.001
40–49 years	20 (10.0%)	26 (13.4%)	46 (11.7%)		0 (0%)	16 (18.4%)	16 (11.4%)	
50–59 years	44 (22.0%)	81 (41.8%)	125 (31.7%)		11 (20.8%)	33 (37.9%)	44 (31.4%)	
60–69 years	77 (38.5%)	69 (35.6%)	146 (37.1%)		21 (39.6%)	28 (32.2%)	49 (35.0%)	
70–79 years	52 (26.0%)	10 (5.2%)	62 (15.7%)		16 (30.2%)	8 (9.2%)	24 (17.1%)	
≥80 years	4 (2.0%)	0 (0%)	4 (1.0%)		4 (7.5%)	0 (0%)	4 (2.9%)	
Median (range) tumor size in the greatest dimension, cm	1.5 (0.4–4.0)	1.75 (0.3–5.5)	1.5 (0.3–5.5)	<0.001	2.0 (0.5–5.0)	1.7 (0.6–3.5)	1.9 (0.5–5.5)	0.601
Tumor size category, *n* (%)								
≤1 cm	53 (26.5%)	30 (15.5%)	83 (21.1%)	<0.001	7 (13.2%)	12 (13.8%)	19 (13.6%)	0.569
>1–2 cm	111 (55.5%)	99 (51.0%)	210 (53.3%)		27 (50.9%)	41 (47.1%)	68 (48.6%)	
>2	35 (17.5%)	65 (33.5%)	100 (25.4%)		19 (35.8%)	32 (36.8%)	51 (36.4%)	
Unknown	1 (0.5%)	0 (0%)	1 (0.3%)		0 (0%)	2 (2.3%)	2 (1.4%)	
Tumor grade category, *n* (%)								
Grade 1	13 (6.5%)	13 (6.7%)	26 (6.6%)	0.255	0 (0%)	3 (3.4%)	3 (2.1%)	0.302
Grade 2	105 (52.5%)	93 (47.9%)	198 (50.3%)		23 (43.4%)	40 (46.0%)	63 (45.0%)	
Grade 3	49 (24.5%)	64 (33.0%)	113 (28.7%)		24 (45.3%)	32 (36.8%)	56 (40.0%)	
Unknown	33 (16.5%)^2^	24 (12.4%)^3^	57 (14.5%)		6 (11.3%)^4^	12 (13.8%)^5^	18 (12.9%)	
Histology, *n* (%)								
IDC	166 (83.0%)	168 (86.6%)	334 (84.8%)	0.187	45 (84.9%)	78 (89.7%)	123 (87.9%)	0.673
ILC	28 (14.0%)	18 (9.3%)	46 (11.7%)		5 (9.4%)	7 (8.0%)	12 (8.6%)	
Micropapillary	0 (0%)	0 (0%)	0 (0%)		1 (1.9%)	0 (0%)	1 (0.7%)	
Mucinous/colloid	3 (1.5%)	7 (3.6%)	10 (2.5%)		1 (1.9%)	1 (1.1%)	2 (1.4%)	
Other/unknown	3 (1.5%)	1 (0.5%)	4 (1.0%)		1 (1.9%)	1 (1.1%)	2 (1.4%)	
Nodal status, *n* (%)								
N0	200 (100%)	194 (100%)	394 (100%)	NA	0 (%)	0 (%)	0 (%)	0.482
N1mi	0 (%)	0 (%)	0 (%)		15 (28.3%)	27 (31.0%)	42 (30.0%)	
1 positive LN	0 (%)	0 (%)	0 (%)		23 (43.4%)	42 (48.3%)	65 (46.4%)	
2 positive LN	0 (%)	0 (%)	0 (%)		12 (22.6%)	11 (12.6%)	23 (16.4%)	
3 positive LN	0 (%)	0 (%)	0 (%)		3 (5.7%)	7 (8.0%)	10 (7.1%)	

*IDC* invasive ductal carcinoma, *ILC* invasive lobular carcinoma.

^1^Comparing CT-treated and untreated patients using chi-square test for categorical parameters and the Wilcoxon Rank Sum test for the continuous parameters.

^2^27/33 (81.8%) of unknown tumor grade were ILC.

^3^16/24 (66.7%) of unknown tumor grade were ILC.

^4^5/6 (83.3%) of unknown tumor grade were ILC.

^5^7/12 (58.3%) of unknown tumor grade were ILC.

**Fig. 2:**
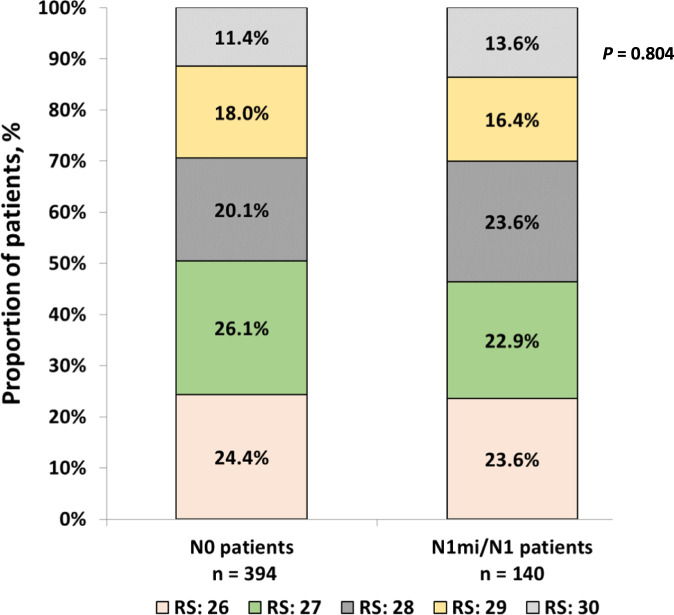
RS distribution in the N0 and N1mi/N1 patients (*p* = 0.804 for comparing RS distribution between N0 and N1mi/N1 patients, chi-square test). RS Recurrence Score.

**Fig. 3:**
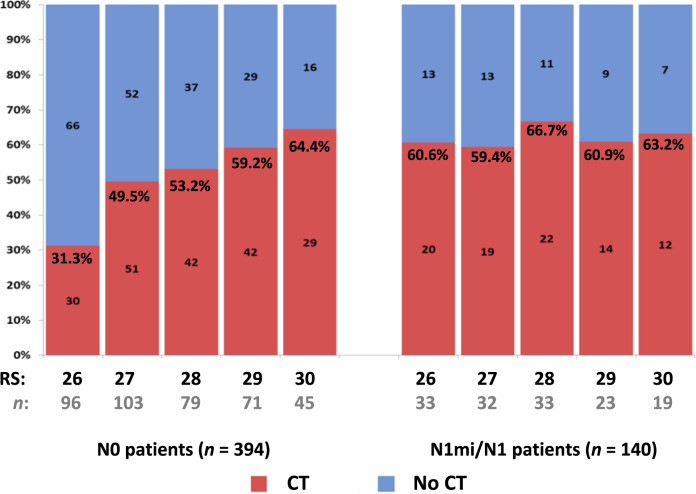
Proportion of patients undergoing CT use by RS result for N0 and N1mi/N1 patients. Number of patients and rates of CT use (%) are displayed on the bar chart. CT chemotherapy, RS Recurrence Score.

RS distribution in the N0 and N1mi/N1 patients was overall similar (*p* = 0.804). In both N0 and N1mi/N1 patients, the RS 26, 27, and 28 groups included each approximately a fifth to a quarter of the patients and the smallest group was the RS 30 patients (Fig. 2). In the N0 patients, the use of adjuvant CT increased with each unit of RS increase (from 31.3% among N0 patients with RS 26 to 64.4% among N0 patients with RS 30). This increase with each RS unit was not observed in the N1mi/N1 patients where the CT use was overall similar within the RS 26–30 range (59.4–66.7%) (Fig. 3).

In a multivariable logistic regression analysis that modeled the odds ratios (OR) of receiving CT as a function of age, grade, tumor size, histology, and RS result in the N0 subgroup, younger age, larger tumor, and higher RS results were significantly associated with higher odds of receiving CT. In a similar analysis on the N1mi/N1 subgroup, the only statistically significant variable was age, with younger age associated with higher odds of receiving CT (Table 2).

**Table 2:** Odds ratios for receiving CT.

Variable	Odds ratio per variable unit (95% Wald confidence limit)	*p*-value^1^
N0 subgroup		
Age at diagnosis, years	0.92 (0.90-0.95)	<0.001
Tumor size, cm	1.75 (1.30-2.34)	<0.001
RS result, unit	1.45 (1.22-1.72)	<0.001
N1mi/N1 subgroup		
Age at diagnosis, years	0.90 (0.86-0.94)	<0.001

^1^*P*-value (2-sided) was derived from the logistic regression analysis (performed on the N0 and N1mi/N1 subgroups separately).

### Clinical outcomes in N0 patients

**Fig. 4:**
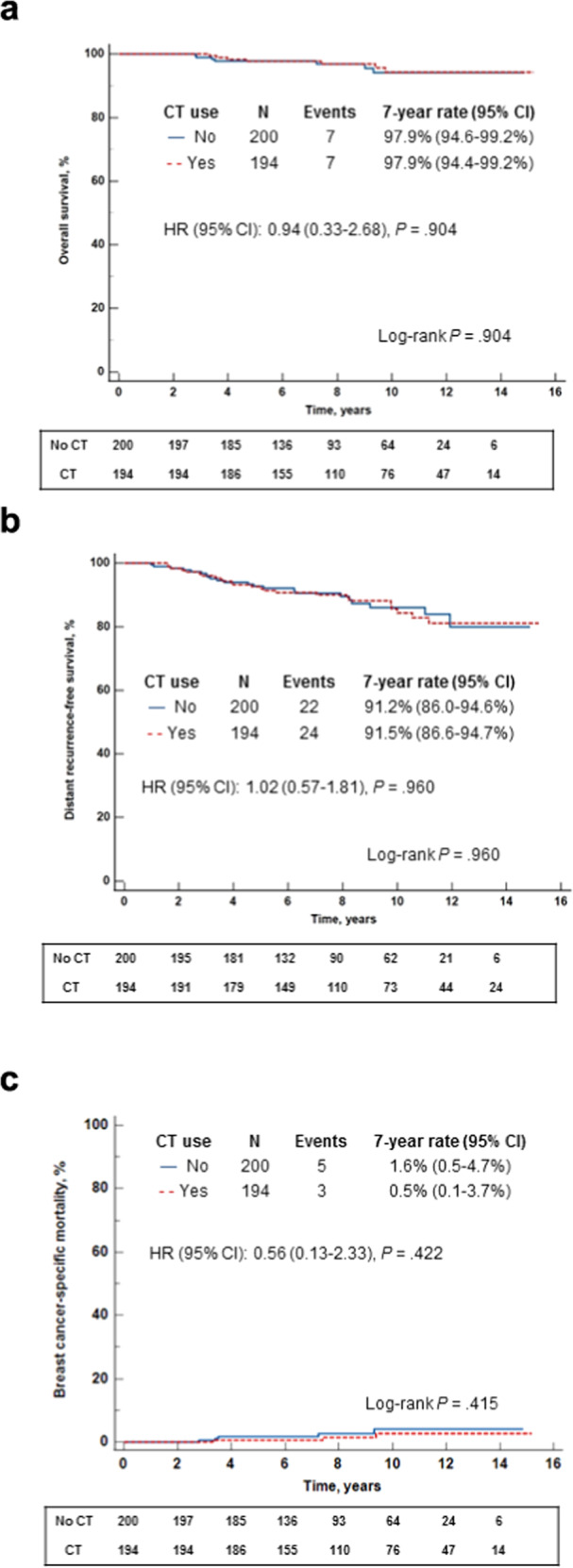
Kaplan-Meier curves for N0 patients by treatment received. **a**. Overall survival; **b**. Distant recurrence-free survival; **c**. Breast cancer-specific mortality. The box under each graph presents the number of patients at risk at each time point. One-degree of freedom log-rank *p*-values were calculated from all the data.

Overall, with a median (interquartile range [IQR]) follow up time of 7.8 (5.8–11.1) years, 40 distant recurrence events, 8 BC-specific deaths, and 6 deaths of other causes were reported. Kaplan-Meier (KM) estimates for overall survival (OS), DRFS, and BC-specific mortality (BCSM) were not statistically significantly different between CT-treated (194 patients, of whom 1 [0.5%] did not receive ET and for 13 [6.7%] ET information was not available) and untreated patients (200 patients, of whom 2 [1.0%] did not receive ET and for 19 [9.5%] ET information was not available). The 7-year rates (95% confidence intervals [CI]) in the CT-treated vs the untreated patients, were 97.9% (94.4–99.2%) vs 97.9% (94.6–99.2%) for OS, 91.5% (86.6–94.7%) vs 91.2% (86.0–94.6%) for DRFS, and 0.5% (0.1–3.7%) vs 1.6% (0.5–4.7%) for BCSM (Fig. 4a–c). Similarly, none of the hazard ratios (HR) for these endpoints (CT vs no CT) were statistically significant. For OS, the HR (95% CI) was 0.938 (0.329-2.675), *p* = 0.904; for DRFS, it was 1.015 (0.568–1.812), *p* = 0.960; and for BCSM, 0.556 (0.133–2.329), *p* = 0.422. In a univariate analysis performed to evaluate the association between distant recurrence and age, tumor grade, tumor size, the RS result and CT use, only tumor size was found to be a statistically significant parameter (HR [95% CI] for >2 cm vs ≤2 cm, 2.12 [1.13–3.99], *p* = 0.020; HR [95% CI] for tumor size as a continuous parameter per cm, 1.55 [1.13–2.06], *p* = 0.0043). All other parameters including treatment with CT were nonsignificant (Supplementary Table 1).

### Clinical outcomes in N1mi/N1 patients

**Fig. 5:**
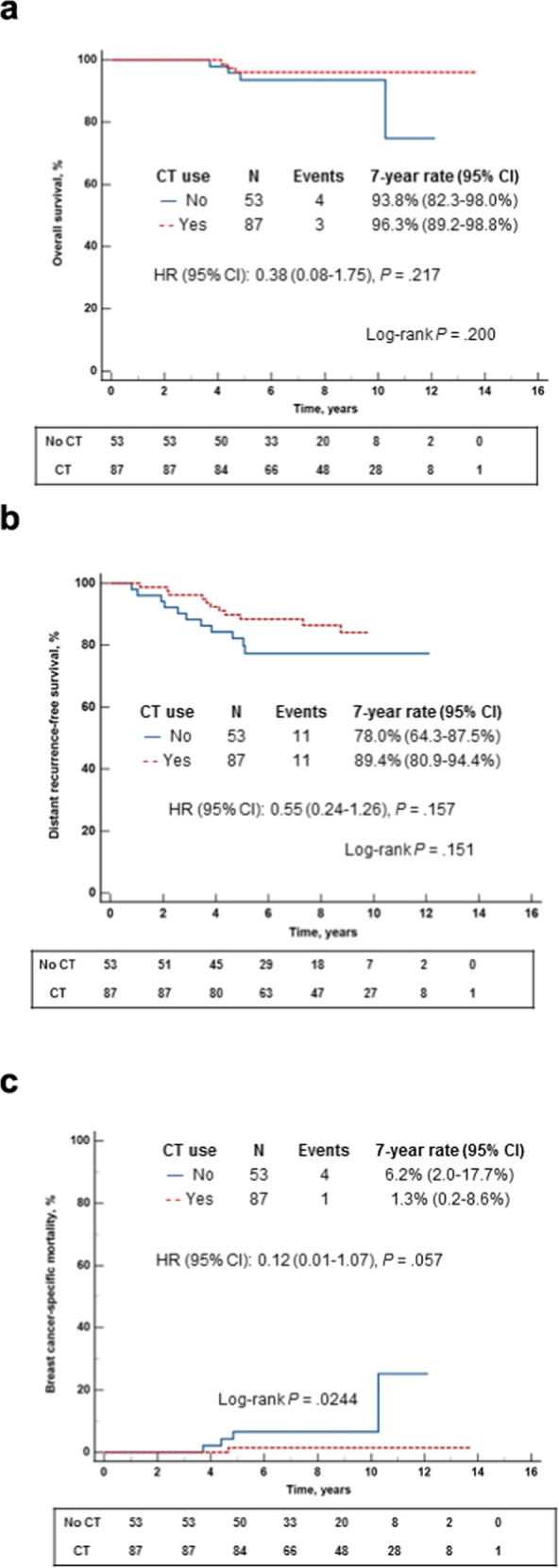
Kaplan-Meier curves for N1mi/N1 patients by treatment received. **a**. Overall survival; **b**. Distant recurrence-free survival; **c**. Breast cancer-specific mortality The box under each graph presents the number of patients at risk at each time point. One-degree of freedom log-rank *p*-values were calculated from all the data.

Overall, with a median (IQR) follow up time of 8.2 (5.8–11.1) years, 20 distant recurrence events, 5 BC-specific deaths, and 2 deaths of other cause were reported. KM estimates were not statistically significantly different between the CT-treated (87 patients, of whom 1 [1.1%] did not receive ET) and untreated patients (53 patients, all of whom received ET) for OS and DRFS. The 7-year rates (95% CI) in the CT-treated vs untreated patients were 96.3% (89.2–98.8%) vs 93.8% (82.3–98.0%) for OS, and 89.4% (80.9–94.4%) vs 78.0% (64.3–87.5%) for DRFS. Notably, the BCSM KM estimates differed significantly between the treatment groups (*p* = 0.024, log-rank test); the 7-year BCSM rate (95% CI) was 1.3% (0.2–8.6%) in CT-treated patients and 6.2% (2.0–17.7%) in the untreated patients (Fig. 5a–c). The HR (CT vs no CT) for the OS and DRFS were not statistically significant. For OS, the HR (95% CI) was 0.384 (0.084–1.754), *p* = 0.217; and for DRFS, it was 0.546 (0.237–1.262), *p* = 0.157. The HR for BCSM, the endpoint where the KM estimates differed significantly demonstrating better outcomes with CT treatment, approached, but did not reach the prespecified threshold for statistical significance: HR (95% CI), 0.115 (0.012–1.070), *p* = 0.057. In a univariate analysis performed to evaluate the association between distant recurrence and age, tumor grade, tumor size, the RS result, and CT use, all the evaluated parameters were nonsignificant (Supplementary Table 2). Notably, the association between distant recurrence and CT use trended towards statistical significance (HR [95% CI] for no CT use vs CT use, 2.24 [0.93–5.43], *p* = 0.0725) (Supplementary Table 2).

## Discussion

In this analysis of RS 26–30 patients in the mature CHS registry, CT rates were higher in N1mi/N1 than N0 patients. For N0 patients, younger age, larger tumor sizes, and higher RS (within this range) were significantly associated with higher odds of receiving CT, whereas for N1mi/N1 patients, only younger age had a significant impact on the odds of receiving CT, suggesting that for N0, but not N1mi/N1 patients, clinicians considered the RS as a continuous parameter (within this range). In N0 patients, clinical outcomes (OS, DRFS, and BCSM) were similar in CT-treated and untreated patients; however, in N1mi/N1 patients, CT-treated patients had statistically significantly better 7-year BCSM, but not OS or DRFS, rates. The absolute difference between CT-treated to untreated patients was 2.5% and 11.4% for 7-year OS and DRFS respectively, however, this difference did not reach statistical significance. Likewise, a univariate analysis on N1mi/N1 patients identified a trend towards statistical significance for the association between CT use and lower risk of distant recurrence. Of note, the N1mi/N1 subgroup was relatively small, and a larger sample size could have potentially identified differences in OS and DRFS as well.

CT use of 49% and 62% for N0 and N1mi/N1 patients, respectively, as observed in the current study is consistent with prior analyses of the CHS registry (50%, and 67%, respectively)^12,13^, as well as analyses of the Surveillance, Epidemiology, and End Results (SEER) data and the National Cancer Data Base (NCDB) (45–58% for N0 patients, 66% for mixed population)^14–18^. Also, in studies where the odds of receiving CT in this RS range were explored, younger age and larger tumor size were found to be associated with higher odds of receiving CT, as in our study. However, unlike the SEER analysis, in the current study, grade 3 was not found to be associated with significantly increased odds of receiving CT, possibly due to the small number of patients with grade 3 disease^14,17,18^.

Thus far, only a few studies focused on clinical outcomes in RS 26–30 patients, and all were real-world retrospective studies^12–19^. Our findings are consistent with the original validation studies where CT benefit (freedom from distant recurrence) in N0 patients was observed in those with RS ≥ 31, and not in those with RS 18–30, although a recent re-analysis of the NSABP B-20 prospective-retrospective study in HER2‒ patients did find CT benefit in RS > 25 patients^8,9,20^. The current findings are also consistent with our previous analyses of the CHS registry which demonstrated no CT benefit with respect to distant recurrence risk in N0 RS 26–30 patients, and suggested some benefit in N1mi/N1 RS 26–30 patients, albeit with a shorter follow-up and a smaller number of patients, as the CHS registry was less mature then^12,13^. Analyses of SEER and NCDB data, which focused on RS 26–30 patients and examined OS and BC-specific survival, did find CT benefit in the RS 26–30 range (for node-negative and node-positive patients); however, this overall benefit was more pronounced in patients with high-risk clinicopathological characteristics such as younger age at diagnosis or high-grade tumors^14–17^. Notably, in the current study, N0 patients who received CT had more high-risk clinicopathologic characteristics than untreated N0 patients, which could explain why no overall CT benefit was observed. Combined, these findings highlight the complexity of adjuvant CT decisions in the intermediate RS range, and the need to further individualize treatment decisions by integrating clinicopathologic characteristics, using a tool such as the recently developed and validated RSClin^21^.

In the current analysis, reflecting real-life clinical practice in a heterogenous patient population with RS 26–30, N0 patients who did not receive CT (e.g., those considered by the clinicians to be low-risk according to clinicopathologic characteristics, but also cases where the patients were offered CT but declined) had excellent clinical outcomes, which are unlikely to be improved by CT. Thus, our study suggests that for some N0 patients with low-risk disease characteristics and RS 26–30, adjuvant CT may be safely omitted. For N1mi/N1 patients with RS 26–30, our data suggest that adjuvant CT may confer some clinical benefit.

The strengths of our study include its representation of real-life clinical practice on a national level (no exclusions based on age, gender, comorbidities, etc), its long follow-up, and the availability of distant recurrence and mortality data. The study is limited by its retrospective non-randomized study, which led to a significant imbalance between CT-treated and untreated patients with respect to baseline patient/tumor characteristics. Also, the number of patients in specific subgroups (e.g., node-positive who were <50 years at diagnosis) was small and prohibited further analyses.

In conclusion, in this real-life data analysis focusing on estrogen receptor (ER) + HER2‒ BC patients with RS 26–30, N0 patients with low-risk according to clinicopathologic characteristics were more likely to forego adjuvant CT, and their clinical outcomes (OS, DRFS, BCSM) with ET alone were excellent, and not statistically significantly different from those of N0 patients who did receive CT. In N1mi/N1 patients, however, CT was associated with a statistically significant reduction in BCSM (an effect on OS and DRFS was also observed but was nonsignificant). Additional retrospective analyses investigating distance recurrence/BCSM in the RS 26–30 range in subgroups by clinicopathologic characteristics are warranted.

## Methods

### Study design and patients

This exploratory retrospective analysis of the prospectively designed CHS registry included all N0/N1mi/N1 ER + BC patients who underwent 21-gene testing through CHS between 1/2006 and 12/2016 and had RS results of 26–30. Exclusion criteria included metastatic disease at the time of testing, diagnosis of breast or other solid malignancy in the 5 years preceding the testing, having 2 tests performed at the same time with one of the RS results >30, and HER2 positivity.

The study was approved by the institutional review board (IRB) of the CHS community division and the participating centers and was granted a waiver for obtaining informed consent due to its retrospective design. The study was conducted in accordance with the declaration of Helsinki.

### Data sources

The Oncotest database was used for RS results and patient/tumor characteristics. Patients’ medical records were used to obtain data on treatments received and clinical outcomes.

### Statistical considerations

Descriptive statistics were used to summarize clinicopathologic characteristics for patients who received CT vs those who did not by nodal status. The characteristics of CT-treated and untreated patients were compared using chi-square test for categorical parameters and the Wilcoxon Rank Sum test for continuous parameters. Multivariable logistic regression was used to determine OR and 95% CI for receiving CT in N0 and N1mi/N1 patients separately. The RS result, tumor size, and age were included as continuous variables; grade and histology were included as categorical variables. The tests and 95% CI on the ORs were Wald-based. KM analyses by CT use were performed for N0 and N1mi/N1 separately. Seven-year estimates and 95% CI were determined for OS, DRFS, and BCSM. Patients without recurrence were censored at the time of last follow up, date of medical records review, or time of death (due to any cause). For BCSM analysis, patients who died with metastatic disease were considered events, and recurrences were ignored. The log-rank test calculated from all the data was used to compare OS, DRFS, and BCSM between CT-treated and untreated patients. HRs and 95% CI were determined using a Cox regression model. Cox regression univariate analysis was used to evaluate the association between distant recurrence and prognostic baseline factors, the RS result, and CT use. JMP^®^ Version 16 (SAS Institute Inc., Cary, NC) was used for the analysis. All tests were 2-sided. *p* ≤ 0.05 was considered statistically significant.

### Reporting summary

Further information on research design is available in the Nature Portfolio Reporting Summary linked to this article.

## Supplementary information

Supplementary materials

Reporting summary form

## Data Availability

The datasets generated during and/or analyzed during the current study are available from the corresponding author upon reasonable request.
