# Supplementary material for: Clinical outcomes in estrogen receptor-positive early-stage breast cancer patients with Recurrence Score 26-30: observational real-world cohort study
Source: NPJ Breast Cancer. 2023 Jun 2;9:49. doi: 10.1038/s41523-023-00549-8 (PMC10238504; doi:10.1038/s41523-023-00549-8)
Supplement: Supplementary file 1 — Supplementary materials [file 41523_2023_549_MOESM1_ESM.pdf]

SUPPLEMENTARY INFORMATION

Supplementary Table 1. Univariate analysis evaluating the association between the variables and distant recurrence on the N0 patient subgroup.

Variable	Comparison	Hazard Ratio (95% confidence intervals)	*P*-value
**Age**			
	≥50 vs <50 years	0.95 (0.40-2.26)	0.906
	As a continuous variable, per year	1.01 (0.98-1.05)	0.462
**Size**			
	>2 vs ≤2 cm	2.12 (1.13-3.99)	0.020
	As a continuous variable, per cm	1.55 (1.13-2.06)	0.0043
**Grade**			
	3 vs 1 or 2	1.42 (0.69-2.89)	0.338
**RS result**			
	As a continuous variable, per unit	0.92 (0.72-1.16)	0.471
**Treatment**			
	No CT vs CT	1.08 (0.58-2.01)	0.811

CT *chemotherapy*, RS *Recurrence Score.*

Supplementary Table 2. Univariate analysis evaluating the association between the variables and distant recurrence on the N1mi/N1 patient subgroup.

Variable	Comparison	Hazard Ratio (95% confidence intervals)	*P*-value
**Age**			
	≥50 vs <50 years	1.34 (0.31-5.79)	0.693
	As a continuous variable, per year	1.01 (0.96-1.05)	0.734
**Size**			
	>2 vs ≤2 cm	1.22 (0.50-2.99)	0.660
	As a continuous variable, per cm	0.87 (0.47-1.49)	0.641
**Grade**			
	3 vs 1 or 2	1.62 (0.65-4.03)	0.300
**RS result**			
	As a continuous variable, per unit	1.05 (0.76-1.45)	0.751
**Treatment**			
	No CT vs CT	2.24 (0.93-5.43)	0.0725

CT *chemotherapy*, RS *Recurrence Score.*
