# Supplementary material for: Clinical outcomes in estrogen receptor-positive early-stage breast cancer patients with Recurrence Score 26-30: observational real-world cohort study
Source: NPJ Breast Cancer. 2023 Jun 2;9:49. doi: 10.1038/s41523-023-00549-8 (PMC10238504; doi:10.1038/s41523-023-00549-8)
Supplement: Supplementary file 2 — Reporting summary form [file 41523_2023_549_MOESM2_ESM.pdf]

nature portfolio

Corresponding author(s): Ofer Rotem

Last updated by author(s): April 21, 2023

Reporting Summary

Nature Portfolio wishes to improve the reproducibility of the work that we publish. This form provides structure for consistency and transparency in reporting. For further information on Nature Portfolio policies, see our Editorial Policies and the Editorial Policy Checklist.

Statistics

For all statistical analyses, confirm that the following items are present in the figure legend, table legend, main text, or Methods section.

n/a	Confirmed	
☐	☒	The exact sample size (*n*) for each experimental group/condition, given as a discrete number and unit of measurement
☒	☐	A statement on whether measurements were taken from distinct samples or whether the same sample was measured repeatedly
☐	☒	The statistical test(s) used AND whether they are one- or two-sided<br>*Only common tests should be described solely by name; describe more complex techniques in the Methods section.*
☐	☒	A description of all covariates tested
☐	☒	A description of any assumptions or corrections, such as tests of normality and adjustment for multiple comparisons
☐	☒	A full description of the statistical parameters including central tendency (e.g. means) or other basic estimates (e.g. regression coefficient) AND variation (e.g. standard deviation) or associated estimates of uncertainty (e.g. confidence intervals)
☒	☐	For null hypothesis testing, the test statistic (e.g. F, t, r) with confidence intervals, effect sizes, degrees of freedom and P value noted<br>*Give P values as exact values whenever suitable.*
☒	☐	For Bayesian analysis, information on the choice of priors and Markov chain Monte Carlo settings
☒	☐	For hierarchical and complex designs, identification of the appropriate level for tests and full reporting of outcomes
☒	☐	Estimates of effect sizes (e.g. Cohen's *d*, Pearson's *r*), indicating how they were calculated

Our web collection on statistics for biologists contains articles on many of the points above.

Software and code

Policy information about availability of computer code

Data collection	Not relevant
Data analysis	Software used for statistical analysis: JMP® Version 16 (SAS Institute Inc., Cary, NC)

For manuscripts utilizing custom algorithms or software that are central to the research but not yet described in published literature, software must be made available to editors and reviewers. We strongly encourage code deposition in a community repository (e.g. GitHub). See the Nature Portfolio guidelines for submitting code & software for further information.

Data

Policy information about availability of data

All manuscripts must include a data availability statement. This statement should provide the following information, where applicable:

- Accession codes, unique identifiers, or web links for publicly available datasets
- A description of any restrictions on data availability
- For clinical datasets or third party data, please ensure that the statement adheres to our policy

The datasets generated during and/or analyzed during the current study are available from the corresponding author on reasonable request.

Human research participants

Policy information about studies involving human research participants and Sex and Gender in Research.

Reporting on sex and gender	The study included no trans women or trans men. Therefore the reporting by sex and gender is the same in this case. As this is a breast cancer study, the number of men/males in the cohort was very small, and no analysis by sex/gender was conducted.
Population characteristics	There were 394 N0 patients and 140 N1mi/N1 patients. Relevant characteristics were described in N0 and N1mi/N1 patients separately and by treatment (CT vs no CT). These included age, grade, tumor size, tumor histology, and the Recurrence Score results. These characteristics are summarized in Table 1 of the manuscript.
Recruitment	The cohort included all N0/N1mi/N1 ER+ breast cancer patients who underwent 21-gene testing through Clalit Health Services between 1/2006 and 12/2016 and had RS 26-30. Exclusion criteria included metastatic disease at the time of testing, diagnosis of breast or other solid malignancy in the 5 years preceding the testing, having 2 tests performed at the same time with one of the RS results >30, and HER2 positivity.
Ethics oversight	The study was approved by the institutional review board (IRB) of the CHS community division and the participating centers.

Note that full information on the approval of the study protocol must also be provided in the manuscript.

Field-specific reporting

Please select the one below that is the best fit for your research. If you are not sure, read the appropriate sections before making your selection.

☒ Life sciences ☐ Behavioural & social sciences ☐ Ecological, evolutionary & environmental sciences

For a reference copy of the document with all sections, see nature.com/documents/nr-reporting-summary-flat.pdf

Life sciences study design

All studies must disclose on these points even when the disclosure is negative.

Sample size	The cohort included all N0/N1mi/N1 ER+ breast cancer patients who underwent 21-gene testing through Clalit Health Services between 1/2006 and 12/2016 and had RS 26-30. The timeframe was chosen as 1/2006 was the approval of the assay by Clalit in Israel, and 12/2016 was chosen to ensure a follow up of at least 5 years when the data were collected and analysed.
Data exclusions	Patients were exlcluded if they had metastatic disease at the time of testing, had diagnosis of breast or other solid malignancy in the 5 years preceding the testing, had 2 tests performed at the same time with one of the RS results >30, or if they were HER2+.
Replication	Not relevant. Real-world data.
Randomization	Not relevant. Real-world data.
Blinding	Not relvant. Observational real-world data.

Reporting for specific materials, systems and methods

We require information from authors about some types of materials, experimental systems and methods used in many studies. Here, indicate whether each material, system or method listed is relevant to your study. If you are not sure if a list item applies to your research, read the appropriate section before selecting a response.

Materials & experimental systems

n/a	Involved in the study
☒	☐ Antibodies
☒	☐ Eukaryotic cell lines
☒	☐ Palaeontology and archaeology
☒	☐ Animals and other organisms
☒	☐ Clinical data
☒	☐ Dual use research of concern

Methods

n/a	Involved in the study
☒	☐ ChIP-seq
☒	☐ Flow cytometry
☒	☐ MRI-based neuroimaging
